# Ox-LDL-mediated ILF3 overexpression in gastric cancer progression by activating the PI3K/AKT/mTOR signaling pathway

**DOI:** 10.18632/aging.204051

**Published:** 2022-05-04

**Authors:** Danping Sun, Mingxiang Zhang, Meng Wei, Zhaoyang Wang, Wen Qiao, Peng Liu, Xin Zhong, Yize Liang, Yuanyuan Chen, Yadi Huang, Wenbin Yu

**Affiliations:** 1 Department of Gastrointestinal Surgery, General Surgery, Qilu Hospital, Cheeloo College of Medicine, Shandong University, Jinan 250012, China; 2 The Key Laboratory of Cardiovascular Remodeling and Function Research, Chinese Ministry of Education, Chinese Ministry of Health and Chinese Academy of Medical Sciences, Department of Cardiology, Qilu Hospital, Cheeloo College of Medicine, Shandong University, Jinan 250012, China; 3 Department of Nursing Department, Qilu Hospital, Cheeloo College of Medicine, Shandong University, Jinan 250012, China

**Keywords:** gastric cancer, dyslipidemia, interleukin-enhancer binding factor 3 (ILF3), ox-LDL, PI3K/AKT/mTOR signaling pathway, biomarkers

## Abstract

Background: This study aimed to investigate the relationship of dyslipidemia and interleukin-enhancer binding factor 3 (ILF3) in gastric cancer, and provide insights into the potential application of statins as an agent to prevent and treat gastric cancer.

Methods: The expression levels of ILF3 in gastric cancer were examined with publicly available datasets such as TCGA, and western blotting and immunohistochemistry were performed to determine the expression of ILF3 in clinical specimens. The effects of ox-LDL on expression of ILF3 were further verified with western blot analyses. RNA sequencing, Kyoto Encyclopedia of Genes and Genomes (KEGG), Gene Ontology (GO), and Gene Set Enrichment Analysis (GSEA) pathway analyses were performed to reveal the potential downstream signaling pathway targets of ILF3. The effects of statins and ILF3 on PI3K/AKT/mTOR signaling pathway, cell proliferation, cell cycle, migration and invasion of gastric cancer cells were investigated with Edu assay, flow cytometry and transwell assay.

Results: Immunohistochemistry and western blot demonstrated that the positive expression rates of ILF3 in gastric cancer tissues were higher than adjacent mucosa tissues. The ox-LDL promoted the expression of ILF3 in a time-concentration-dependent manner. ILF3 promoted the proliferation, cell cycle, migration and invasion by activating the PI3K/AKT/mTOR signaling pathway. Statins inhibited the proliferation, cell cycle, migration and invasion of gastric cancer by inhibiting the expression of ILF3.

Conclusions: These findings demonstrate that ox-LDL promotes ILF3 overexpression to regulate gastric cancer progression by activating the PI3K/AKT/mTOR signaling pathway. Statins inhibits the expression of ILF3, which might be a new targeted therapy for gastric cancer.

## INTRODUCTION

Gastric cancer (GC) is a major health burden worldwide, especially in Eastern and Western Asia [1]. Besides, the incidence of GC ranks second in China [2]. Due to the lack of obvious symptoms, specific clinical, imaging, or pathological manifestations, patients are usually diagnosed at the advanced stage [3, 4]. Despite the development in surgery, chemotherapy, immunotherapy, and targeted therapy, the overall survival rate and prognosis remain unsatisfactory in patients with GC, with a poor 5-year survival rate (<30%) [5, 6]. Among them, the metastasis and recurrence of GC are the main reasons for treatment failure and the patient death [7, 8]. Therefore, it is critical to study the underling molecular of the development of GC, which also can help us identify more molecular markers for monitoring the prognosis of patients.

To date, conventional serum tumor markers are applied in GC screening, including carcinoembryonic antigen (CEA), alpha-fetoprotein (AFP), cancer antigen 19-9(CA19-9), cancer antigen 72-4(CA72-4) and cancer antigen 125(CA125), and are also used in the predicting of the prognosis, recurrence, or metastasis [9–11]. However, as a result of the lack of sensitivity and specificity, these biomarkers are not recommended for GC detection and prognostic follow-up [12].

The successful eradication of Helicobacter pylori (Hp) effectively lowered the morbidity of distal GC, while the number of gastric cancers at the esophagogastric junction and upper third was increasing year by year, and new cases were gradually showing a younger trend [13]. Studies found that obesity contributes greatly to the occurrence of proximal GC [14]. Obesity is a disorder of energy balance and abnormal lipid metabolism, which is mainly characterized with the upregulated blood concentration of low-density lipoprotein cholesterol (LDL-C) [15, 16]. LDL-C is delivered throughout the body in the form of LDL. LDL is very prone to be oxidized by reactive oxygen species (ROS) to form oxidized LDL (ox-LDL) [17, 18]. Therefore, excessive ox-LDL indicates the abnormal lipid conditions in obese subjects. It is well-known that ox-LDL negatively impacts on hypertension, atherosclerosis, and cardiovascular diseases [19]. More and more studies demonstrated that the increased ox-LDL was positively associated with cancer development such as colon, breast, and ovarian cancer [20–22]. Some studies have demonstrated that ox-LDL can induce mutagenesis, stimulate proliferation, initiate metastasis, and induce treatment of resistance [23, 24].

Although the occurrence and development of GC involves a wide range of metabolic pathways [25], little has been written about the roles of abnormal lipid metabolism in this regard. Thus, the mechanism remains unclear that how ox-LDL promotes the occurrence and development of GC.

Interleukin-enhancer binding factor 3 (ILF3) is known as NF90/NF110, a member of the double-stranded RNA-binding proteins (DRBPs), which is crucial in RNA metabolism from transcription to degradation, including transcription, translation, maintaining the stability of mRNA and primary microRNA processing [26]. In recent years, ILF3 has been widely studied and been linked to multiple malignant tumors. For example, ILF3 facilitated the occurrence of colorectal cancer by regulating the mRNA stability of serine–glycine–one-carbon (SGOC) SGOC genes [27]. ILF3 overexpression was associated with poor clinical outcome for patients with lung cancer, and ILF3 can also be employed to guide the hierarchical postoperative management of patients with lung cancer [28]. ILF3 might serve as an important promoter in hepatocellular carcinoma proliferation and migration, and could be a potential therapeutic target in hepatocellular carcinoma [29]. ILF3 increased the expression of HIF-1α/VEGFA in cervical cancer cells to promote angiogenesis through PI3K/AKT signaling pathway [30]. Moreover, it has been reported the important function of ILF3 in the development of acquired chemoresistance in GC patients [31].

In addition to contributing to carcinogenesis, ILF3 is also a risk factor for coronary artery disease, venous thromboembolism, and stroke [32]. Studies have reported that ILF3 was associated with the serum concentrations of LDL cholesterol, and has been found to be a candidate gene for myocardial infarction in Japanese individual [33, 34]. The reports on the relationship of ox-LDL and the expression of ILF3, and their relationship with the development and progress of GC have never been reported. Therefore, further research is required to explore the association of ox-LDL and ILF3 and the underlying molecular mechanism in the development of GC.

Statins are 3-hydroxy-3-methylglutaryl coenzyme A (HMG-CoA) reductase inhibitors, which are intensively used for dyslipidemia and cardiovascular disease prevention [35]. Besides, statins use also could reduce total cancer risk and lower cancer-specific mortality [36, 37]. In a large population-based retrospective cohort study, statins use reduced the risk of cancer and cancer-related mortality [38]. A meta-analysis revealed that statins was associated with 32% reduction in the risk of GC [39]. Recent years, *in vivo* and *in vitro* studies have demonstrated that statins may exert anti-cancer effects via a number of potential mechanisms including inhibition of mevalonate pathway, anti-inflammatory and anti-angiogenesis [40, 41], and could lower the cholesterol levels in human gastric cancer cell lines [42].

This is the first study to demonstrate that statins can downregulate the expression of ILF3 in GC treatment by reducing blood lipid levels. Thus, we hypothesized that ox-LDL promoted ILF3 overexpression and promoted proliferation, cell cycle, migration, and invasion of gastric cancer cells via PI3K/AKT/mTOR signaling pathway, and that statins might be the targeted drugs to treat GC in clinical practice.

## RESULTS

### ILF3 was overexpressed in gastric cancer patients

**Figure 1:**
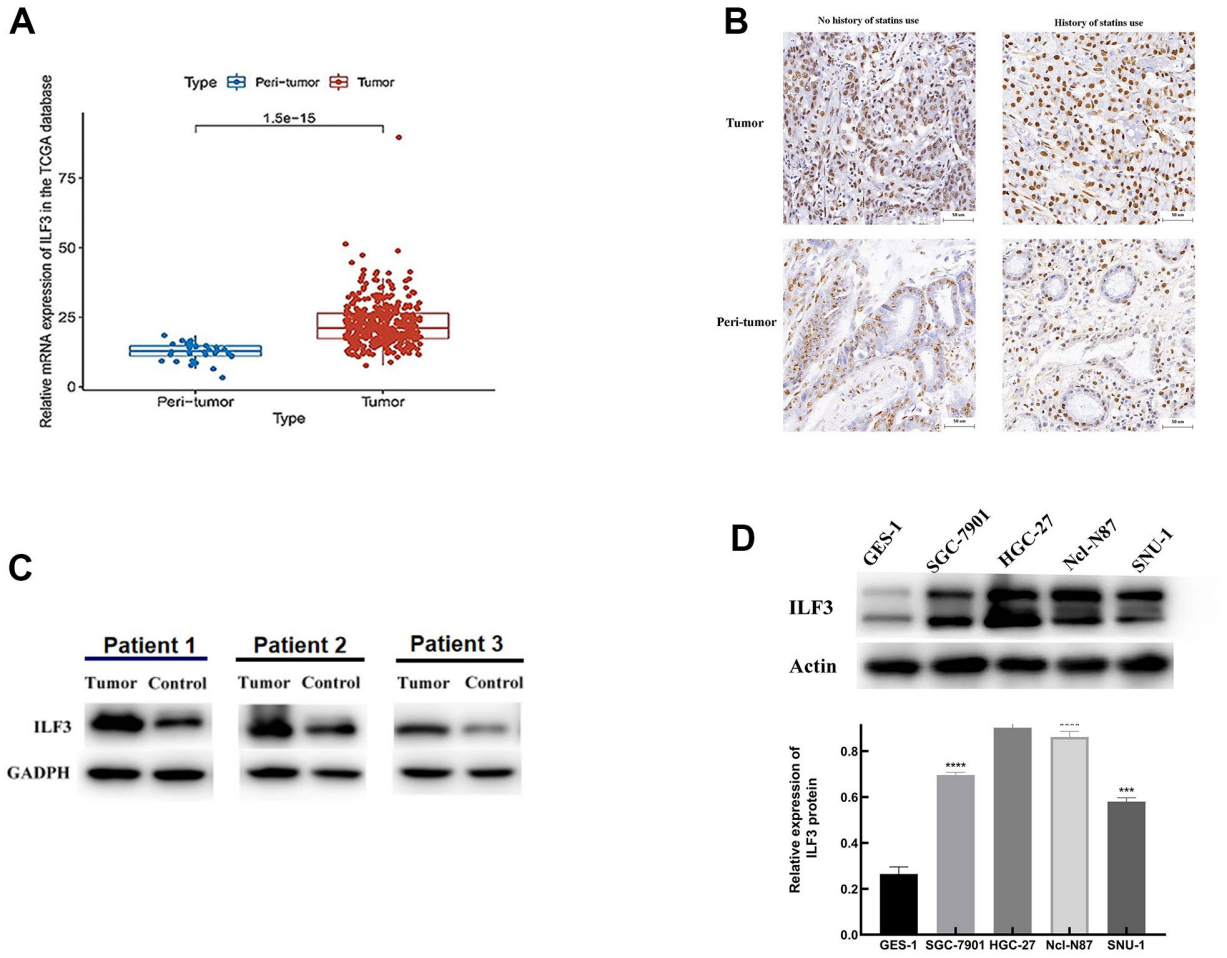
**ILF3 was up-regulated in gastric cancer.** (**A**) Expression of ILF3 mRNA in gastric cancer samples (n = 375) and normal samples (n = 32) from the TCGA data. (**B**) ILF3 protein expression was detected by IHC. ILF3 positive staining in tumor tissues was increased relative to non-cancerous tissues. (**C**) Western blot analysis of ILF3 in paired tumor tissues and non-cancerous tissues. The ILF3 expression level of mRNA was higher in cancer tissue than in normal tissues. (**D**) Western blot analysis of ILF3 protein in gastric epithelial cell line (GES-1) and gastric cancer cells lines (SGC-7901, HGC-27, Ncl-N87, and SNU-1). The ILF3 protein expression was significantly higher in gastric cancer cell lines than gastric epithelial cell line. **P < 0.01, ***P < 0.001, ****P < 0.0001.

To determine the expression level of ILF3 in GC samples, analysis of the mRNA expression data from The Cancer Genome Atlas (TCGA) including 375 GC samples and 32 normal cases demonstrated that the ILF3 expression level was higher in cancer tissue than normal tissues (Figure 1A, P<0.05).

To measure the ILF3 expression levels in clinical GC tissues, immunohistochemical staining of ILF3 was performed in a cohort of 33 human GC tissues and matched adjacent non-cancerous tissues. Patients with GC were divided into subgroups according to use of statins medication. The results revealed that the ILF3 expression in GC was higher than in no-neoplastic tissues. And the expression of ILF3 in patients taking statins was significantly lower than that without taking statins (Figure 1B, P<0.05).

Moreover, the ILF3 protein expression was quantified by western blot. As shown in Figure 1C, ILF3 protein level was significantly upregulated in tissues from GC (P<0.05).

Expression of ILF3 protein was detected in all 4 gastric cancer cell lines (SGC-7901, HGC-27, Ncl-N87, and SNU-1) and gastric epithelial cell lines (GES-1). Western blotting found that GC cell lines expressed higher ILF3 protein than gastric epithelial cell line (GES-1) (Figure 1D, P<0.05). Among the 4 cell lines of GC, the expressions of ILF3 in HGC-27 and Ncl-N87 were remarkedly increased compared with other cell lines. Therefore, Ncl-N87 and HGC-27 cell lines were selected for subsequent experiments.

### Whole-genome RNA-seq analysis of ILF3

**Figure 2:**
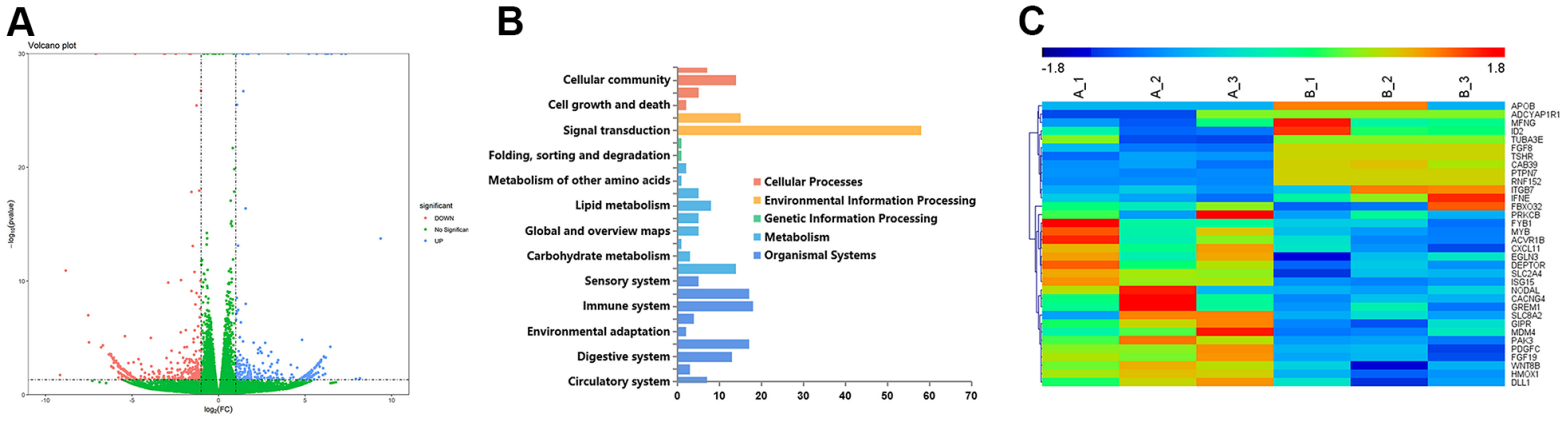
**Volcano plot comparing gene expression between the interference group (infected with ILF3-specific siRNA) and the transfected negative control group in gastric cancer cell SGC-7901, respectively named si-ILF3 group and si-nc group.** (**A**) The abscissa represents the logarithmic value of the fold change(log2FC) of the difference in the expression of a certain gene in the si-ILF3 group and si-nc group. The greater the absolute value of the abscissa, the greater the difference of expression between the two groups. The y-coordinate represents the negative log of p-value, namely the -log10 (p-value). The higher the value of ordinate was, the more the differential expression of genes was reliable. (**B**) The potential important roles that ILF3 played in the gastric cancer cells. (**C**) Genes that were potentially regulated by ILF3 expression in whole-genome RNA sequencing.

Correlation analysis of the whole-genome RNA-sequencing (RNA-seq) of ILF3 was performed with small interference (si-ILF3) to explore potential biological effects of ILF3. The volcano map of transcriptomics analysis showed a global view of gene expression, and showed that ILF3 played important role in processing the environmental and genetic information, the cellular processes, metabolism, and organismal systems (Figure 2A, 2B).

Among all the differentially expressed genes (Supplementary Table 1), APOB and FGF19 were involved in the lipid biosynthetic process. APOB was also involved in the lipoprotein transport. Based on the result, we speculated that ILF3 may participate in the regulation of lipid metabolism by regulating the expression of APOB. Besides, DEPTOR was overexpressed after knocking out ILF3 using ILF3-specific small interference RNA (si-ILF3), and previous studies have reported that DEPTOR is an endogenous mTOR inhibitor [43], whose expression was negatively regulated by mTOR. Therefore, the overexpressed DEPTOR may inhibit the mTOR signaling pathway and thus exerted a tumor suppressor effect (Figure 2C).

### Functional characteristics of ILF3 in gastric cancer cells

**Figure 3:**
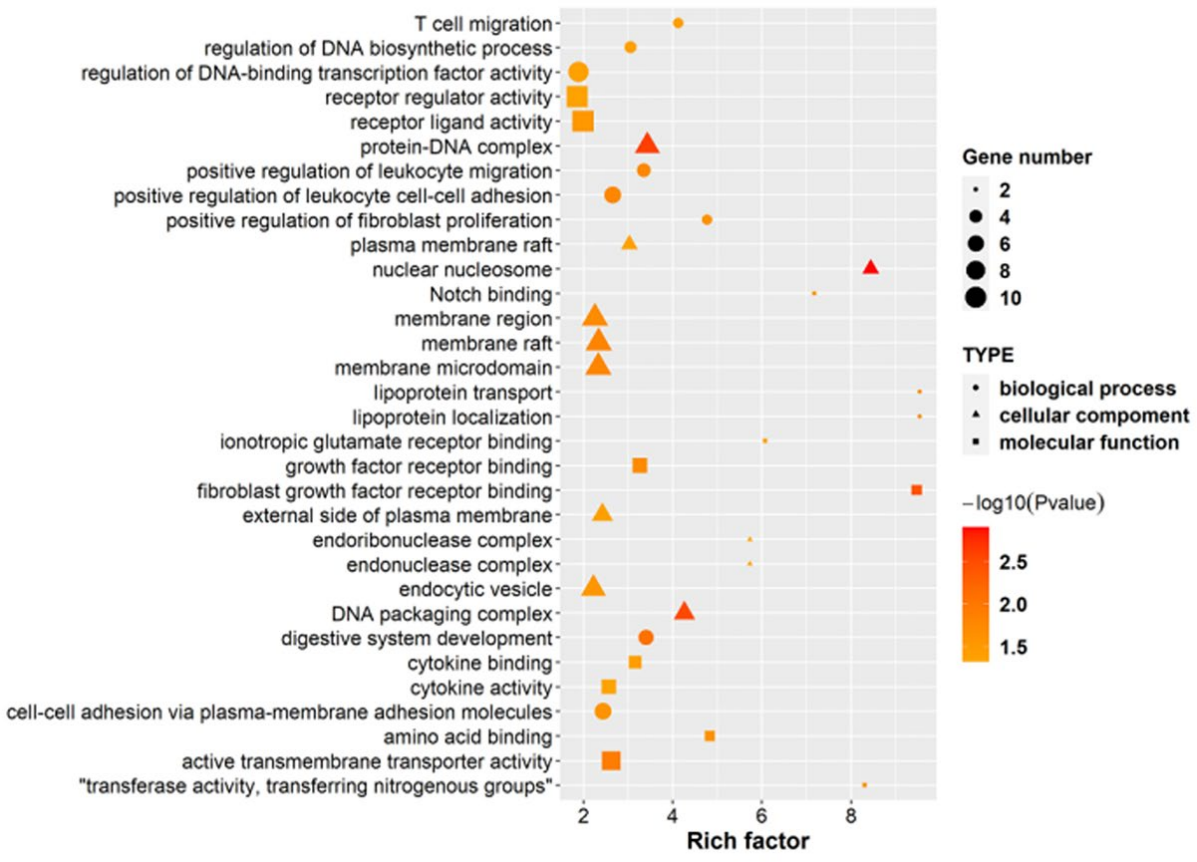
**GO enrichment analysis between the si-ILF3 group and si-nc group in gastric cancer cell SGC-7901.** GO enrichment analysis revealed the functions of ILF3 in gastric cancer cells, including BPs (biological processes), CCs (cellular components), and MFs (molecular function), which showed that ILF3 played important roles in the development of gastric cancer.

GO analysis was applied to reveal the function characteristics of ILF3, including BPs (biological processes), CCs (cellular components), and MFs (molecular function) (Figure 3 and Supplementary Table 2).

For BP analysis, the functions of ILF3 were mainly involved in digestive system development, lipoprotein transport and localization, cell migration (T cell, leukocyte, lymphocyte etc.), cell-cell adhesion, cell proliferation (fibroblast, neural precursor cell, smooth muscle cell etc.), and DNA synthesis and transcription. For example, a number of investigators have proposed the role of lipoproteins in the promotion of cancer progression [44]. And LDL is the largest cholesterol transporter of the body [45]. The LDL receptor on tumor cells is overexpressed to meet the high demand of cholesterol which is necessary for the rapid cell proliferation and de novo membrane synthesis. And ox-LDL is an independent risk factor for GC. Thus, understanding the mechanism and function between ILF3 and ox-LDL facilitates the discovery of new targets for the treatment of GC.

For CC analysis, ILF3 was associated with membrane raft. Membrane rafts of lipid rafts are small, dynamic membrane domains which are enriched with cholesterol and sphingolipids [46]. Membrane rafts do not only occur in the plasma membrane but also in intracellular membranes and extracellular vesicles [47]. Membrane rafts are significant in cellular signal pathways, and regulating cell proliferation, migration, invasion and apoptosis, which are responsible for the initiation, development and progression of malignant tumors [48, 49]. Studies have shown that ox-LDL may destroy the severity of lipid rafts, leading to corresponding pathological processes, and promote the progression of cancer [50]. Further research to link the ox-LDL to impaired lipid raft function due to the interaction with the expression of ILF3 is needed.

For MF analysis, ILF3 was associated with Notch binding. Abnormal Notch signaling is associated with a variety of genetic and acquired disease, including cancers [51]. Notch signaling pathway regulated cellular proliferation and differentiation in a variety of gastrointestinal tract tissues, including the stomach. [52, 53]. Studies have found that mTOR signaling was reduced after Notch inhibition suggesting that mTOR might be downstream of Notch in GC cells [54, 55]. Therefore, ILF3 may eventually activate the mTOR signaling pathway through the Notch signaling to promote the proliferation of GC cells.

### ILF3 involved signaling pathway alterations in gastric cancer cells

**Figure 4:**
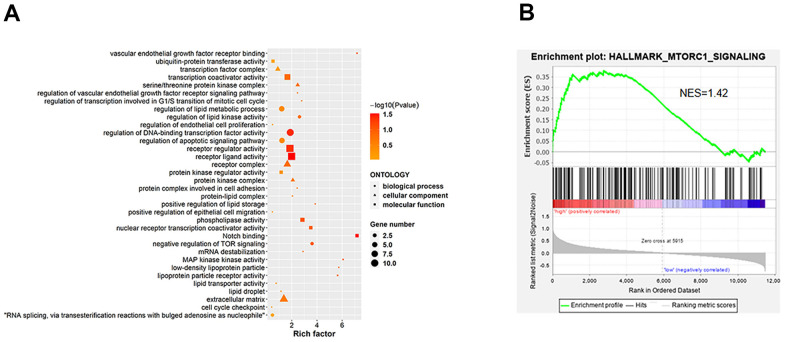
**KEGG enrichment analysis between the si-ILF3 group and si-nc group in gastric cancer cell SGC-7901.** (**A**) KEGG analysis showed the signaling pathways that ILF3 was involved in gastric cancer cell SGC-7901. (**B**) Results of the GSEA showed that ILF3 participated in the regulation of mTOR signaling pathway. NES = normalized enrichment score.

KEGG pathway analysis of ILF3 identified 14 statistically significant signaling pathways, including African trypanosomiasis, Glycine, serine and threonine metabolism, Mineral absorption, Tryptophan metabolism, Arrhythmogenic right ventricular cardiomyopathy (ARVC), Gap junction, Hypertrophic cardiomyopathy (HCM), Systemic lupus erythematosus, Alcoholism, signaling pathways regulating stem cell pluripotency, TGF-beta signaling pathway, Dilated cardiomyopathy (DCM), Glycosaminoglycan biosynthesis - heparan sulfate / heparin and mTOR signaling pathway (Figure 4A and Supplementary Table 3).

To further investigate ILF3-mediated signaling pathways in gastric carcinogenesis, Gene Set Enrichment Analysis (GSEA) was performed, which found that ILF3 was positively associated with upregulation of mTOR signaling pathway. It is well-known that mTOR is an important downstream target of PI3K/AKT. Therefore, we need further experiments to prove that ILF3 can promote the occurrence and development of GC through PI3K/Akt/mTOR signaling pathway (Figure 4B).

### ox-LDL promoted ILF3 overexpression in gastric cancer cells

To study the potential links between ox-LDL and ILF3, we set the concentration gradient of ox-LDL (0, 20, 40, 60, 80, and 100 μg/ml) and the time gradient (24, 48, and 72 h) of stimulation, respectively.

**Figure 5:**
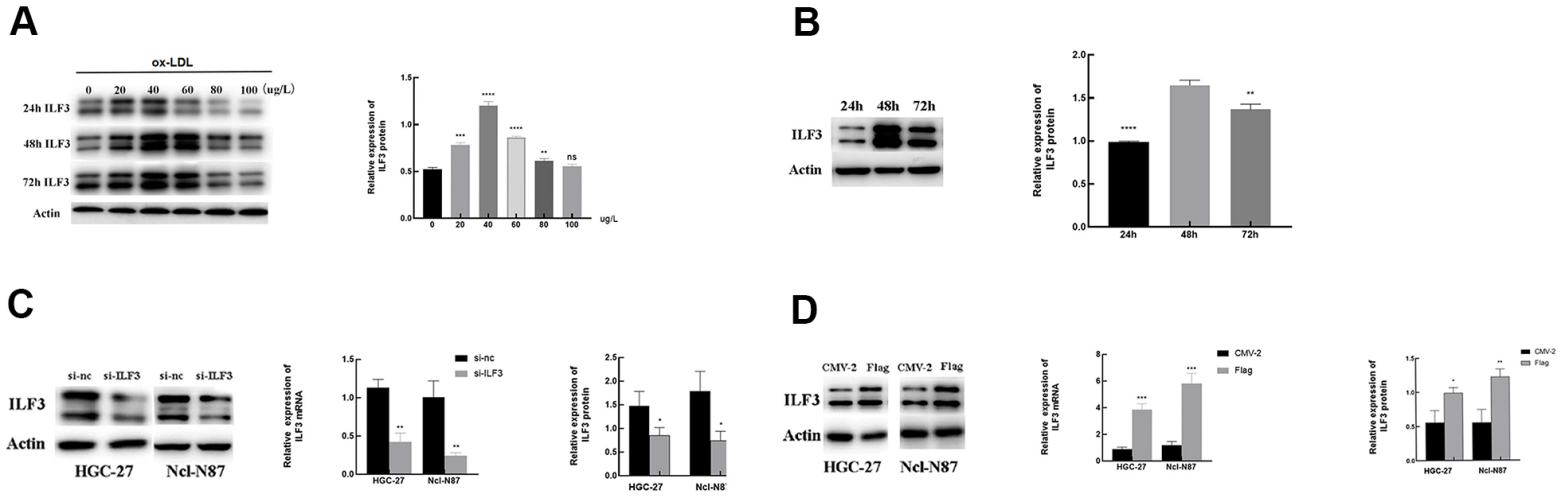
**The relationship of the expression of ILF3 and ox-LDL.** (**A**, **B**) ox-LDL promoted the expression of ILF3 in a time-concentration-dependent manner, the optimal concentration and intervention time was 40 μg/ml and 48h. (**C**) ILF3 was knocked down by ILF3-specific small interference RNA (siRNA) in HGC-27 and Ncl-N87 cells. The mRNA and protein expression level of ILF3 was verified by RT-qPCR and western blot. The ILF3 expression at the mRNA and protein levels was significantly lower in the ILF3-siRNA group compared with the negative control group in HGC-27 and Ncl-N87 cells. (**D**) ILF3 was overexpressed by ILF3-overexpressed plasmids (flag-ILF3) in HGC-27 and Ncl-N87 cells. The mRNA and protein expression level of ILF3 was verified by RT-qPCR and western blot. The ILF3 expression at the mRNA and protein levels was significantly higher in the flag-ILF3 group compared with the negative control group in HGC-27 and Ncl-N87 cells. **P < 0.01, ***P < 0.001, ****P < 0.0001 vs. 0 μg/L or 48 h groups.

Western blotting showed that when the stimulation time was set to 48h, the protein expression of ILF3 was significantly upregulated after ox-LDL stimulation in 20, 40, 60, and 80 μg/ml concentration range compared with blank control group without ox-LDL stimulation. The protein expression of ILF3 was highest when the concentration of ox-LDL was 40 μg/ml. When the concentration of ox-LDL is 100ug/ml, the protein expression of ILF3 is not statistically significant compared with control group (Figure 5A, P<0.05). Therefore, an ox-LDL concentration of 40 μg/ml was used for all subsequent experiments.

When the concentration of ox-LDL was set to 40 μg/ml, the expression of ILF3 was measured by western blot at 24h, 48h and 72h after stimulation. The expression of ILF3 was the highest when the stimulation time wea 48h and it was statistically significant (Figure 5B, P<0.05).

To conducted loss-of-function assays to detected the effect of ILF3 on GC cells, ILF3 was knocked down with ILF3-specific small interference RNA (si-ILF3). Compared to the negative control (si-nc) group, mRNA and protein levels of ILF3 were lower (Figure 5C, P<0.05). Also, ILF3 was overexpressed with ILF3-overexpressed plasmids (flag-ILF3). Compared to the vector plasmids (CMV2) group, the ILF3 expression was significantly higher (Figure 5D, P<0.05).

### ILF3 promoted proliferation, cell cycle, migration, and invasion of gastric cancer cells, and statins may exert an anti-tumor effect by inhibiting ILF3 expression in gastric cancer

Combined with BP (biological processes) analysis, to verify the role of ILF3 in promoting GC, *in vitro* experiments were conducted.

**Figure 6:**
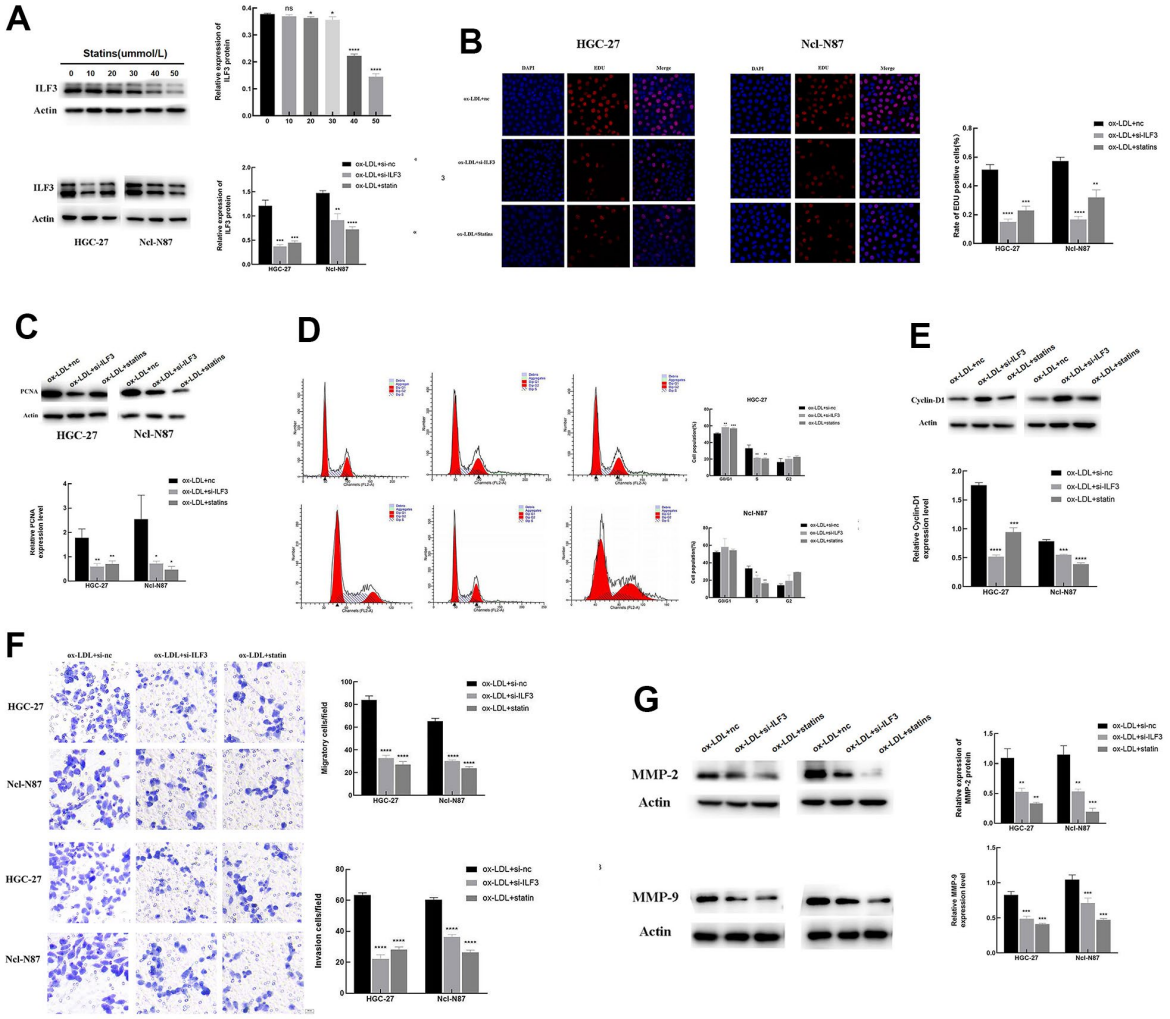
**ILF3 promotes gastric cancer cells proliferation, cell cycle, migration, and invasion, and statins may exert an anti-tumor effect by inhibiting ILF3 expression in gastric cancer.** (**A**) Statins inhibited the expression of ILF3 in a concentration-dependent manner, the optimal concentration was 40&mu;mol/L. The protein expression of ILF3 was significantly downregulated with ILF3-specific small interference RNA (si-ILF3) and statin treatment compared to control group. The expression level of ILF3 was analyzed with western blot. (**B**) Edu assay analyzed the effects of ILF3 on cell proliferation of gastric cancer cells. ILF3-specific small interference RNA (si-ILF3) and statins treatment inhibited the proliferation of HGC-27 and Ncl-N87 cells compared to control group. (**C**) The protein expression of PCNA was lower expressed in the ILF3-specific small interference and statins treatment groups compared to control group. (**D**) Flow cytometry analyzed the effect of ILF3 on cell cycle of gastric cancer cells. ILF3-specific small interference RNA (si-ILF3) and statins treatment inhibited the cell cycle of HGC-27 and Ncl-N87 cells compared to control group. (**E**) The protein expression of cyclin-D1 was lower expressed in the ILF3-specific small interference and statins treatment groups compared to control group. (**F**) Transwell assay analyzed the effect of ILF3 on cell migration and invasion. ILF3-specific small interference RNA (si-ILF3) and statins treatment inhibited the migration and invasion of HGC-27 and Ncl-N87 cells compared to control group. (**G**) The protein expression of MMP-2 and MMP-9 were lower expressed in the ILF3-specific small interference and statins treatment groups compared to control group. **P < 0.01, ***P < 0.001, ****P < 0.0001.

To further reveal the possible relationship between statins and ILF3, we set the concentration gradient of statins (0, 10, 20, 30, 40, and 50 &mu;mol/L) to stimulate GC cells. Western blot assay showed that the expression of ILF3 was decreased with the increase in concentration of statins. Follow-up experiments chose 40 &mu;mol/L as the treatment concentration of statins treatment. And western blot analysis also found that ILF3-specific small interference RNA (si-ILF3) downregulated the protein expressions of ILF3 compared with blank control group (Figure 6A, P<0.05). To reveal the role of ILF3 and the feasibility of statins in the treatment of GC, the changes of cell phenotype was analyzed after treatment with ox-LDL + si-nc, ox-LDL+ILF3-specific small interference RNA (si-ILF3) and ox-LDL + statins. And we set the ox-LDL + si-nc group as control group.

The effect of ILF3 on cell proliferation was evaluated by Edu assay (Figure 6B). The percentage of Edu-positive GC cells in the ILF3-specific small interference and statins treatment groups were lower than that in the control group. The PCNA expression was lower expressed in the ILF3-specific small interference and statins treatment groups compared to control (Figure 6B, 6C, P<0.05).

Flow cytometry analyzed the effect of ILF3 on cell cycle of GC cells. Down-expression of ILF3 significantly increased the proportion of HGC-27 in the G0/G1 phase, and inhibited the proportion of HGC-27 in the S phase of cell cycle in the ILF3-specific small interference and statins treatment groups compared to control group. Down-expression of ILF3 significantly inhibited the proportion of Ncl-N87 in the S phase of cell cycle in the ILF3-specific small interference and statins treatment groups compared to control group, but the proportion of Ncl-N87 in the G0/G1 phase was not statistically significant(Figure 6D, P<0.05). Western blot analysis showed that the protein expression of cyclin-D1, the marker of G1-S phase transition, was lower expressed in the ILF3-specific small interference and statins treatment groups compared to control group(Figure 6E, P<0.05).

The role of ILF3 on cell cycle was analyzed by flow cytometry. Inhibiting ILF3 expression significantly upregulated the proportion of HGC-27 and Ncl-N87 in the G0/G1 phase, and inhibited the percentage of HGC-27 and Ncl-N87 in the S phase in the ILF3-specific small interference and statins treatment groups compared to control group (Figure 6D, P<0.05). As shown in Figure 6E, compared to control group, the protein level of cyclin-D1, the marker of G1-S phase transition, was lower expressed in the ILF3-specific small interference and statins treatment groups (P<0.05).

Cell invasion and migration modulated by ILF3 were assessed by transwell assay. Less invasive and migrated GC cells were found in the ILF3-specific small interference and statins treatment groups compared to control group (Figure 6F, P<0.05). The protein levels of MMP-2 and MMP-9 were lower expressed in the ILF3-specific small interference and statins treatment groups compared to control (Figure 6G, P<0.05).

### Knockdown of ILF3 inhibited the activity of PI3K/AKT/mTOR signaling pathway in gastric cancer cells

**Figure 7:**
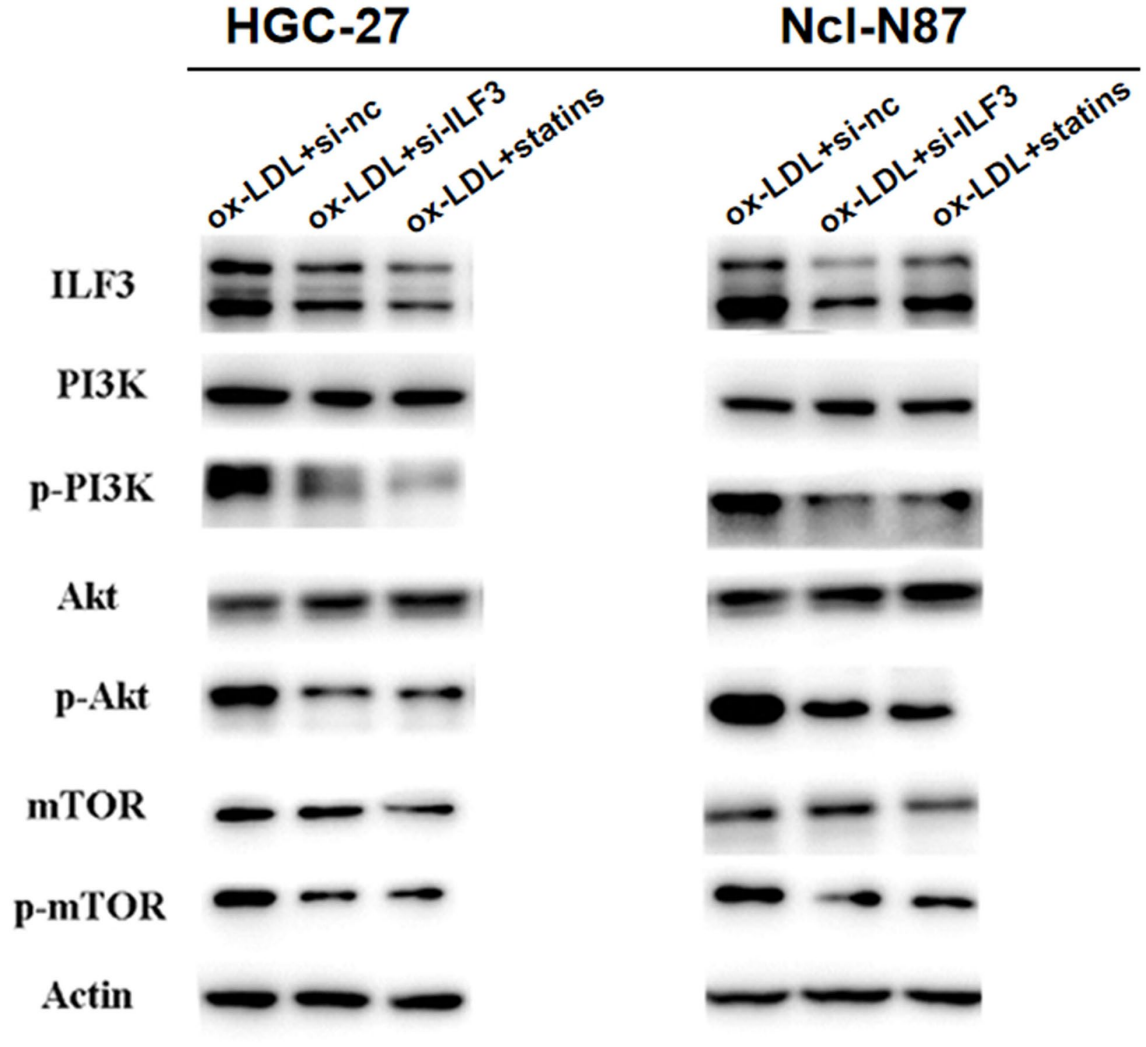
**ILF3 was involved in the regulation of PI3K/AKT/mTOR signaling pathway.** The effect of ILF3 on PI3K/AKT/mTOR signaling pathway was verified with western blot. ILF3-specific small interference RNA (si-ILF3) and statins treatment significantly inhibited PI3K/AKT/mTOR signaling pathway. The expression of p-PI3K/PI3K, p-AKT/AKT, and p-mTOR/mTOR were significantly downregulated compared to control group. And the expression of PI3K, AKT and mTOR and p-mTOR did not change significantly.

KEGG and GSEA analyses revealed that ILF3 affected GC through PI3K/AKT/mTOR signaling pathway. Western blot analysis of p-PI3K/PI3K, p-AKT/AKT, and p-mTOR/mTOR found that p-PI3K, p-AKT, and p-mTOR were significantly downregulated ILF3-specific small interference and statins treatment groups compared to control group. And the expression of PI3K, AKT and mTOR did not change significantly (Figure 7). These findings demonstrated that ILF3 regulated the proliferation, cell cycle, migration and invasion by activating the PI3K/AKT/mTOR signaling pathway.

### ILF3 promoted gastric cancer cell proliferation, cell cycle, migration and invasion via PI3K/AKT/mTOR signaling pathway

To further define the role of PI3K/AKT/mTOR signaling pathway affected by ILF3 in the regulation of GC cell proliferation, cell cycle, migration, and invasion, the malignant biological of GC cells overexpressing ILF3 treated with PI3K/AKT inhibitor LY294002. ILF3-overexpressed plasmids (flag-ILF3) or vector plasmids (CMV2) were transfected into GC cells HGC-27 and Ncl-N87. The changes of cell phenotype were analyzed after treatment with ox-LDL+CMV2, ox-LDL+flag-ILF3, ox-LDL+flag-ILF3+PI3K/AKT inhibitor LY294002. And we set the ox-LDL+flag-ILF3 as the control group.

**Figure 8:**
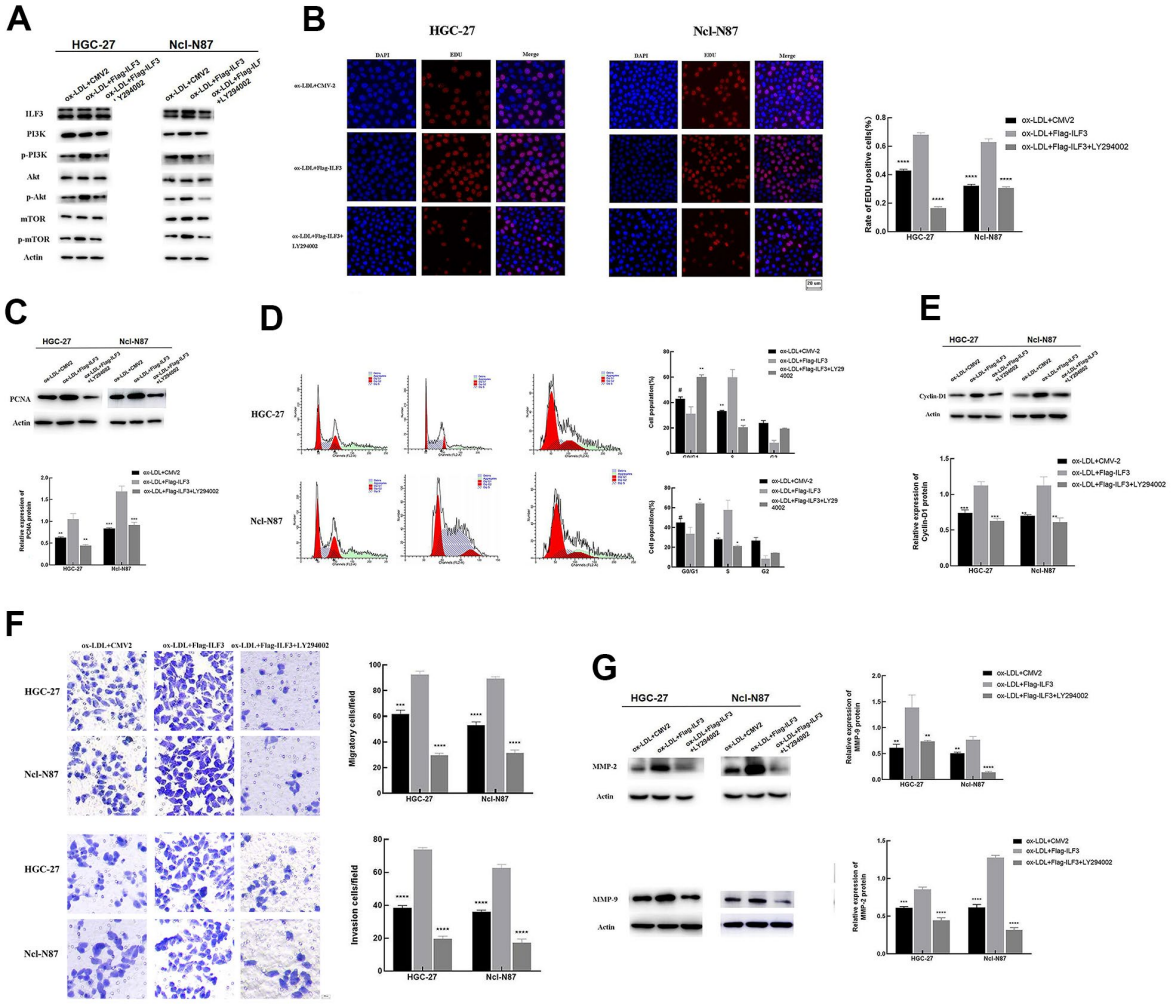
**ILF3 regulated gastric cancer cell proliferation, cell cycle, migration, and invasion via PI3K/AKT/mTOR signaling pathway.** (**A**) The effect of ILF3 on PI3K/AKT/mTOR signaling pathway was verified with western blot. ILF3-overexpressed plasmids (flag-ILF3) treatment significantly activated PI3K/AKT/mTOR signaling pathway. The expression of p-PI3K/PI3K, p-AKT/AKT, and p-mTOR/mTOR were significantly upregulated compared to vector plasmids (CMV2) group. And PI3K/AKT inhibitor LY294002 treatment significantly inhibited the PI3K/AKT/mTOR signaling pathway. (**B**) Edu assay analyzed the effects of PI3K/AKT/mTOR signaling pathway affected by ILF3 on cell proliferation of gastric cancer cells. ILF3-overexpressed plasmids (flag-ILF3) treatment significantly promoted the proliferation of HGC-27 and Ncl-N87 cells. And PI3K/AKT inhibitor LY294002 treatment significantly inhibited the proliferation of HGC-27 and Ncl-N87 cells compared to control group ILF3-overexpressed plasmids (flag-ILF3) group. (**C**) The protein expression of PCNA was lower expressed in the PI3K/AKT inhibitor LY294002 treatment and vector plasmids (CMV2) groups compared to ILF3-overexpressed plasmids (flag-ILF3) group. (**D**) Flow cytometry analyzed the effects of PI3K/AKT/mTOR signaling pathway affected by ILF3 on cell cycle of gastric cancer cells. ILF3-overexpressed plasmids (flag-ILF3) treatment significantly promoted the cell cycle of HGC-27 and Ncl-N87 cells. And PI3K/AKT inhibitor LY294002 treatment significantly inhibited the cell cycle HGC-27 and Ncl-N87 cells compared to ILF3-overexpressed plasmids (flag-ILF3) group. (**E**) The protein expression of cyclin-D1 was lower expressed in the PI3K/AKT inhibitor LY294002 treatment and vector plasmids (CMV2) groups compared to ILF3-overexpressed plasmids (flag-ILF3) group. (**F**) Transwell assay analyzed the effects of PI3K/AKT/mTOR signaling pathway affected by ILF3 on migration and invasion of gastric cancer cells. ILF3-overexpressed plasmids (flag-ILF3) treatment significantly promoted the migration and invasion of HGC-27 and Ncl-N87 cells. And PI3K/AKT inhibitor LY294002 treatment significantly inhibited the migration and invasion HGC-27 and Ncl-N87 cells compared to ILF3-overexpressed plasmids (flag-ILF3) group. (**G**) The protein expression of MMP-2 and MMP-9 were lower expressed in the PI3K/AKT inhibitor LY294002 treatment and vector plasmids (CMV2) groups compared to ILF3-overexpressed plasmids (flag-ILF3) group. **P < 0.01, ***P < 0.001, ****P < 0.0001.

Western blotting found that p-PI3K, p-AKT, and p-mTOR were downregulated in ILF3-vector plasmids (CMV2) and PI3K/AKT inhibitor LY294002 treatment groups compared to ILF3-overexpressed plasmids (flag-ILF3) group. And the expression of PI3K, AKT and mTOR did not change significantly (Figure 8A). These findings demonstrated that ILF3 activated the PI3K/AKT/mTOR signaling pathway. And the inhibition of the signaling pathway could reverse the gastric cancer-promoting effect of the overexpression of ILF3.

Edu assay analyzed the effects of PI3K/AKT/mTOR signaling pathway affected by ILF3 on cell proliferation of GC cells. The proportion of Edu-positive GC cells was higher than in the ILF3-overexpressed plasmids group compared to control group. LY294002 treatment reduced the proportion of Edu-positive GC cells compared to the ILF3-overexpressed plasmids group (Figure 8B, P<0.05). The LY294002 treatment decreased the expression of PCNA compared to the ILF3-overexpressed plasmids group (Figure 8C, P<0.05). Overall, inhibition of the signaling pathway impeded ILF3-mediated the proliferation of GC cells.

Flow cytometry analyzed the effects of the signaling pathway affected by ILF3 on the cell cycle. ILF3 overexpression downregulated the proportion of HGC-27 and Ncl-N87 in the G0/G1 phase and increased the proportion of HGC-27 and Ncl-N87 in the S phase in the ILF3-overexpressed plasmids group compared to control group. LY294002 treatment increased the proportion of HGC-27 and Ncl-N87 in the G0/G1 phase and reduced the proportion of HGC-27 and Ncl-N87 in the S phase compared to the ILF3-overexpressed plasmids group (Figure 8D, P<0.05). The LY294002 treatment downregulated the cyclin-D1 expression compared with ILF3-overexpressed group (Figure 8E, P<0.05). Overall, inhibition of the PI3K/AKT/mTOR signaling pathway impeded ILF3-mediated the cell cycle of GC cells.

Transwell assay analyzed the effects of the signaling pathway affected by ILF3. The number of migrated and invasive GC cells was remarkedly elevated in the ILF3-overexpressed plasmids group compared to control group (Figure 4F, P<0.05). LY294002 treatment reduced the number of migrated and invasive GC cells compared to the ILF3-overexpressed plasmids group (Figure 8E, P<0.05). The protein expression of MMP-2 and MMP-9 were higher in ILF3-overexpressed plasmids group compared to control group. LY294002 treatment decreased the expression of MMP-2 and MMP-9 compared to the ILF3-overexpressed plasmids group (Figure 8G, P<0.05). Overall, suppress the PI3K/AKT/mTOR signaling pathway impeded ILF3-mediated the invasion and migration of GC cells.

### ILF3 promoted tumorigenesis of *in vivo* gastric cancer cells

**Figure 9:**
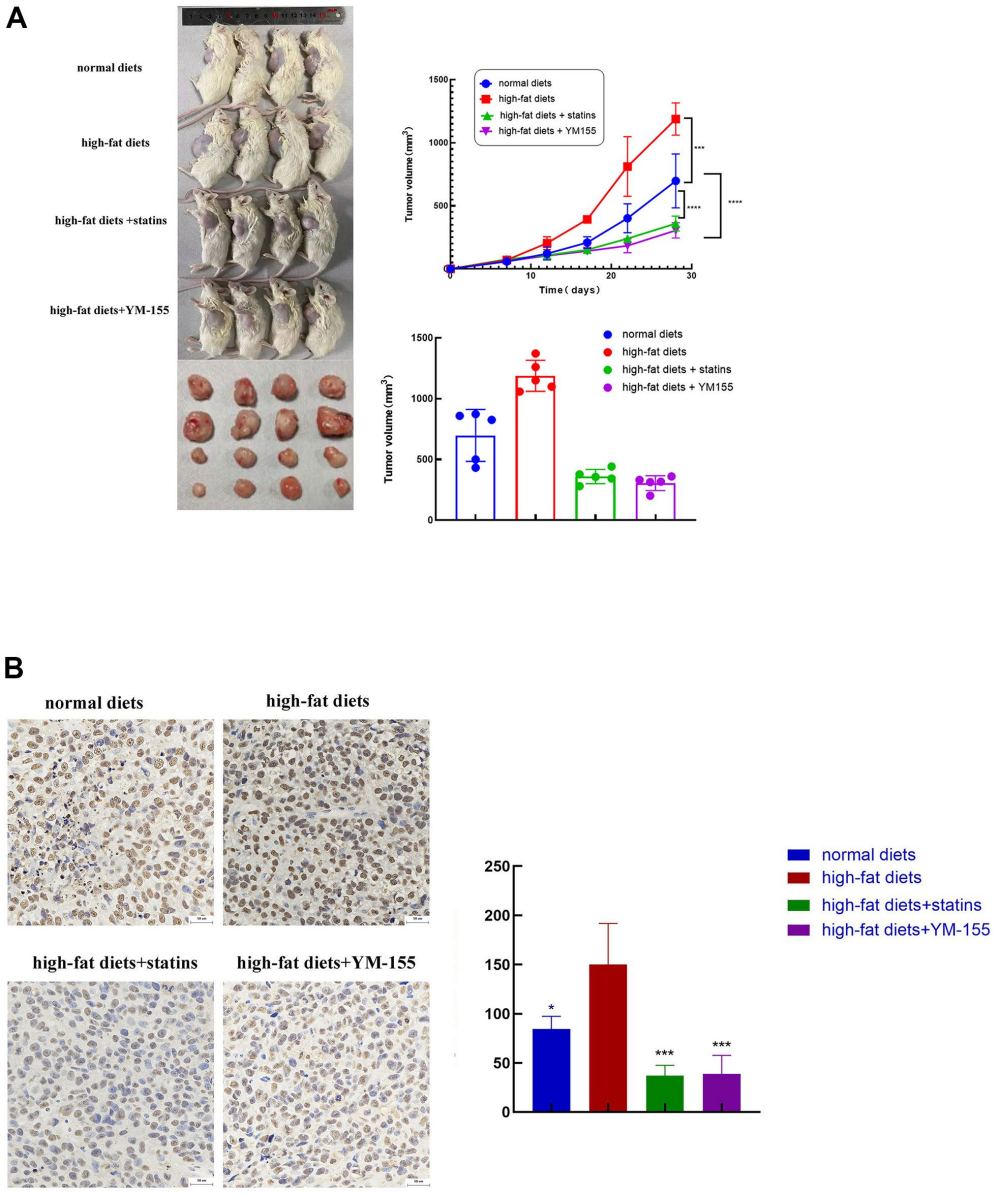
**ILF3 promoted tumor cell growth *in vivo*.** (**A**) Images of nude mouse tumorigenesis test after four weeks of implantation. Comparison of tumor volume between normal diets, high-fat diets, high-fat diets+statin and high-fat diets + ILF3 inhibitor-YM155. Tumors in high-fat diet group were bigger than normal diet group. Tumors in statin and ILF3 inhibitor-YM-155 groups were smaller than high-fat diet group. (**B**) Immunohistochemistry showed that the expression of ILF3 in normal diets, high-fat diets, high-fat diets+statin and high-fat diets + ILF3 inhibitor-YM155 groups. **P < 0.01, ***P < 0.001, ****P < 0.0001.

To test the consequent of ILF3 on the growth of GC cells *in vivo*, a xenograft tumor model was employed. Sixteen of twenty nude mice survived up to the end of the experiments. Tumors of the high-fat diets had larger volumes than those of the normal diets group. Tumors of statins treatment and YM-155 (ILF3 inhibitor) treatment groups had smaller volumes than those of the high-fat diets group (Figure 9A, P<0.05). Immunohistochemistry showed that the expression of ILF3 in high-fat diets was significantly higher compared with the normal diet group. The expression of ILF3 in statins treatment and YM-155 (ILF3 inhibitor) treatment groups were lower than those of the high-fat diets group (Figure 9B, P<0.05).

### ILF3 was involved in the regulation of PI3K/AKT/mTOR signaling pathway in gastric cancer cell SGC-7901

**Figure 10:**
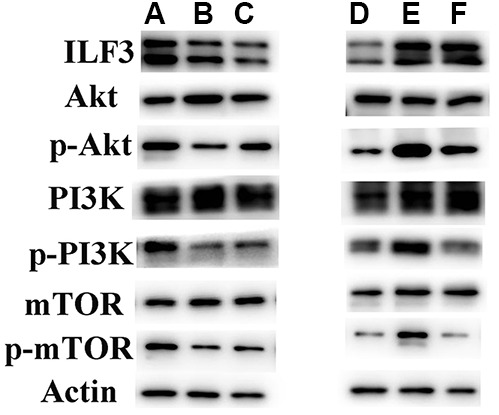
**ILF3 was involved in the regulation of PI3K/AKT/mTOR signaling pathway in gastric cancer cell SGC-7901.** (**A**:ox-LDL+si-nc;**B**:ox-LDL+si-ILF3;**C:**ox-LDL+statin;**D**:ox-LDL+CMV-2;**E**:ox-LDL+flag-ILF3;**F**:ox-LDL+flag-ILF3+PI3K/AKT inhibitor LY294002). (**A**–**C**) ILF3-specific small interference RNA (si-ILF3) and statins treatment significantly inhibited PI3K/AKT/mTOR signaling pathway. The expression of p-PI3K/PI3K, p-AKT/AKT, and p-mTOR/mTOR were significantly downregulated compared to control group. And the expression of PI3K, Akt and mTOR and p-mTOR did not change significantly. (**D**–**F**) ILF3-overexpressed plasmids (flag-ILF3) treatment significantly activated PI3K/AKT/mTOR signaling pathway. The expression of p-PI3K/PI3K, p-AKT/AKT, and p-mTOR/mTOR were significantly upregulated compared to vector plasmids (CMV2) group. PI3K/AKT inhibitor LY294002 treatment significantly inhibited the PI3K/AKT/mTOR signaling pathway.

The experiments were conducted in SGC-7901 cells to confirm the relationship of ILF3 and the related signaling pathway. Western blotting found that p-PI3K, p-AKT, and p-mTOR were significantly downregulated ILF3-specific small interference and statins treatment groups compared to control group. The expression of PI3K, AKT and mTOR did not change significantly. The protein level of p-PI3K, p-AKT, and p-mTOR were significantly downregulated in ILF3-vector plasmids (CMV2) and PI3K/AKT inhibitor LY294002 treatment groups compared to control group. But the expression of PI3K, Akt and mTOR did not obviously changed (Figure 10).

## DISCUSSION

Gastric cancer ranks fifth in the global incidence (5.6%) and fourth in tumor-related mortality (7.7%) [1]. Studies have investigated molecular mechanisms of GC. However, the pathogenesis needs to be further revealed. Therefore, more and more research has focused on patient-specific factors to provide more effective treatment to achieve precise treatment, thereby reducing morbidity and mortality [56]. Recently, studies have shown that lipid metabolism dysfunction plays significant role in gastric carcinogenesis [57], which provides a new research direction to predict, prevent, and early diagnosis of GC. High-fat diets and obesity have been regarded as risk factors of GC [58]. Therefore, dietary modifications and losing weight primary and secondary prevention strategies to reduce the risk of GC. In addition, this study focused on the molecular mechanism and targets of GC caused by abnormal lipid metabolism and find a new biomarker for early diagnoses of GC. In addition to formulating personalized treatment plans for targets, it is more important to find targeted drugs to successfully transform research results into results that benefit patients.

Obesity leads to diseases such as abnormal lipid metabolism and hyperlipidemia [59], and is an important risk factor for carcinogenesis. Some studies have demonstrated that improvement of blood lipid levels and obesity control reduced the occurrence rate of GC, colorectal cancer, and esophageal cancer [60–62]. Abnormal lipid metabolism triggers a cascade of molecular events to finally cause malignancy. So, understanding mechanisms that how obesity or abnormal lipid metabolism induce GC development is important to prevent and treat GC. A case-control study in South Korea has found the higher blood LDL in patients with GCs compared to healthy people [63]. And LDL can easily be oxidized to ox-LDL which is an important feature of abnormal lipid metabolism [64, 65]. While, ox-LDL is critical in the progress of atherosclerosis and non-alcoholic steatohepatitis [66, 67]. Recently, studies have revealed the function of ox-LDL in the cancer cell proliferation [24], cell cycle [68], EMT [69], angiogenesis [70] and metastasis [71]. However, the detailed mechanism of ox-LDL to regulate the downstream gene expression or involved signaling pathways in GC is still unclear. It has been shown that ILF3 was associated with plasma LDL cholesterol, and has been considered as a candidate gene for the patients with acute myocardial infarction in Japanese [33, 34]. But the reports on the relationship of ox-LDL and the expression of ILF3, and their relationship with the occurrence and development of GC have never been reported. We found ox-LDL promoted the expression of ILF3 in a time-concentration dependent manner in GC cells, which provided a new research direction of the relationship of ox-LDL and GC.

Previous research has reported that ILF3 plays as a transcriptional coactivator and involves in proliferation and metastasis of tumor [72]. To further explore the function of ILF3, GO analysis on the sequencing results was performed to guide the next step of phenotyping experiments. GO analysis exhibited that ILF3 was vital in GC cell proliferation, migration and invasion, indicating the oncogenic role of ILF3. We showed that ILF3 promoted proliferation of GC *in vitro*, and ILF3 overexpression accelerated cell proliferation rate with upregulation of PCNA expression in GC cells, suggesting that ILF3 promotes GC progression. Previous research reported that downregulation of ILF3 could delay hepatocellular carcinoma cell cycle progression and inhibit cell proliferation [73]. And to a certain extent, the cell cycle reflects the proliferation status of GC cells. After ILF3 inhibition by ILF3-specific small interference, the proportion of GC cells in G0/G1 phase was increased, which indicated that ILF3 promoted the proliferation and cell cycle of GC cells.

Cell invasion and migration are typical hallmark of malignancies [74]. Consistent with previous study in melanoma, our results indicated that ILF3 could accelerate the invasion and migration of GC cells *in vitro* [75]. When ILF3 was down-regulated by siRNA, the migration and invasion of GC cells *in vitro* were significantly inhibited. Thereby, the results validated that ox-LDL promoted the overexpression of ILF3 to enhance proliferation, cell cycle, migration and invasion of GC cells, thereby promoting the occurrence and development of GC.

Statins can lower plasma cholesterol, and are extensively used to prevent cardiovascular diseases [76, 77]. Currently studies have found that statins have multiple functions, including anti-inflammatory, antioxidant, antithrombotic, anticancer, and cancer chemopreventive effects. Several studies found that statins improved chemosensitivity in a variety of cancers and was used as an adjuvant to chemotherapy [78]. A meta-analysis of studies supported the association between statin and GC risk [39]. A previous study found that Hp infection was the most common cause for GC [39], and cytotoxin-associated gene A (CagA) is the most-important virulence factor of Hp [79]. Hp can manipulate the cholesterol-rich microdomains (also called lipid rafts), which contributes to CagA functions and pathogenesis [80], and statins disrupt the lipid raft of cell membranes to inhibit pathogenic function of CagA [79]. A meta-analysis demonstrated that the inhibition of cancer by statins was more pronounced in distal than proximal GC, and that Hp infection is not the only risk factor for GC [42]. Besides, study had reported that the inhibition of the mevalonate pathway reverted the malignancy potential and reduce the invasiveness of cancers [81]. However, molecular mechanisms for the use of statins to treat GC remain unclear. Thus, it is crucial to find new targets to provide a reliable fundamental basis for statins against GC.

From CC analysis, the results showed that ILF3 was also associated with membrane raft. We proposed that statins may play anti-tumor effects in GC by acting on ILF3. To verify this hypothesis, in the present study we conducted a series of experiments. We found statins treatment significantly inhibited the mRNA and protein expressions of ILF3 compared to control group in GC cells. The cell functional experiments revealed that statins treatment significantly inhibited the proliferation, cell cycle, migration and invasion of GC cells by inhibiting ILF3. The animal experiments revealed that ILF3 inhibitor (YM-155) and statins treatment not only the volume of subcutaneous, but also the expression of ILF3 were significantly reduced. These findings were consistent with previous findings that the expression of ILF3 was significantly lower in patients with taking statins when compared to patients who didn’t take statins. Thereby, these data supported that ILF3 acted as a tumor promoter and served as a potential new target for the use of statins to treat GC patients, and ILF3 elimination is a significant target to prevent and intervent GC, providing rationale for the use of statins to treat GC.

Obesity-associated cancer is a major health burden and has been intensively studied. Researchers have identified multiple cancer risk factors including adipokines, cytokines, insulin/insulin-like growth factor axis, and other cellular signal pathways. Among them, lipids regulate some important oncogenic pathways such as PI3K/AKT/mTOR, Ras, or Wnt pathways [82, 83]. In this study, based on KEGG pathway enrichment analysis and GSEA analysis, the expression levels of ILF3 could regulate the activation of the PI3K/AKT/mTOR signaling pathway The PI3K/AKT/mTOR signaling pathway is important in tumor progression, including cell proliferation, invasion, metastasis, cell cycle, apoptosis, and metabolic functions [84, 85]. Thus, discovery of new specific targets to activate PI3K/AKT/mTOR signaling pathway has become a hotspot of research among targeted interventions of GC. And the role of ILF3 in the modulation of PI3K/AKT/mTOR signaling pathway is unrevealed.

This study validated that interference of ILF3 and statins treatment downregulated the phosphorylation PI3K, AKT and mTOR, thereby inhibit the PI3K/AKT/mTOR signaling pathway in GC cells. The rescue experiments were designed, in which PI3K/AKT/mTOR signaling pathway inhibitors under the premise of overexpression of ILF3 were used to observe the phenotypic changes of GC cells. The rescue experiments demonstrated that the inhibitor of signaling pathway reversed overexpression effect of ILF3 on cell proliferation, cell cycle, migration, and invasion of GC cells. Therefore, ILF3 promoted cell proliferation, cell cycle, migration, and invasion by regulating PI3K/AKT/mTOR signaling pathway in GC cells.

ILF3 and its regulated PI3K/AKT/mTOR signaling pathway are valuable resource for GC in the field of abnormal lipid metabolism, providing insights into lipid metabolism and discovery of energy metabolism-based molecular biomarker pattern and new antitumor targets/drugs to effectively treat GC. ILF3 might be a new GC marker for risk stratification in people with obesity or abnormal lipid level. According to the individual risks, appropriate preventive measures can be taken. For high-risk group of GC, statins treatment improved blood lipid levels when inhibiting the expression of ILF3, and reduced the occurrence of GC. Statins combined with chemotherapy might aid personalized treatment of treatment of high-expressed ILF3 GC patients.

## CONCLUSIONS

This study shows that ox-LDL promotes the expression of ILF3 through the PI3K/AKT/mTOR signaling pathway, thus facilitates the proliferation, cell cycle, migration and invasion of gastric cancer cells, providing a potential new biomarker for the early detection and the therapeutic target of gastric cancer patients.

## MATERIALS AND METHODS

### Clinical specimen acquisition and analysis

The data of mRNA sequencing and corresponding clinical information of 375 GC samples and 32 normal cases were obtained from The Cancer Genome Atlas (TCGA) database (https://tcga-data.nci.nih.gov/). The paired gastric cancer tissue and matched adjacent normal tissue were collected from 33 patients with GC admitted to Qilu Hospital, Cheeloo College of Medicine, Shandong University (Jinan, China) between January 2020 and December 2020 before surgery, these patients did not receive any other treatments, such as radiotherapy, chemotherapy, and targeted therapy. Informed consent was obtained from all participants.

### Whole-genome RNA sequencing

The gastric cancer cells SGC-7901 were randomly divided into the interference group (infected with ILF3-specific siRNA) and the transfected negative control group. RNeasy mini kit (Qiagen, Germany) was applied to isolate total RNA. TruSeq™ RNA Sample Preparation Kit (Illumina, USA) was used to synthesize paired-end libraries. The final cDNA library was then created with PCR by purification and enrichment. The library construction and sequencing were implemented by Sinotech Genomics Co., Ltd (Shanghai, China). R package edgeR was carried out to analyze the mRNA differential expression. Differentially expressed RNAs with |log2(fold-change)| value >1 and q value <0.05 were reserved for further analysis.

### Functional enrichment analyses

We performed the Gene Ontology (GO) [86] and Kyoto Encyclopedia of Genes and Genomes (KEGG) pathway enrichment analyses based on the RNA sequencing, which included the biological processes (BP), cellular components (CC), molecular functions (MF), and pathways. The p value < 0.05 were considered significant.

 Gene Set Enrichment Analysis (GSEA) was performed to figure out the signaling pathway which may be regulated by ILF3. The normalized enrichment score (|NES|) > 1 was considered as statistical significance.

### Cell lines and cell culture

The gastric cancer cell lines GES, SGC-7901, HGC-27, Ncl-N87, and SNU-1 were obtained from Fuheng company, Shanghai, China, and were cultured in 1640 media (Gibco) supplemented with 10% fetal bovine serum (Gibco) and 1% penicillin/streptomycin (Millipore) in a humidified incubator with 5% CO2 at 37° C.

### ILF3-siRNA transfection and groupings

For the knockdown of ILF3, cells were transfected with ILF3-specific siRNA or scramble siRNA with lipofectamine 3000 reagents. Each targeting sequence was shown: si-ILF3: 5’-ACG UGA CAC GUU CGG AGA ATT-3’; si-nc: 5’-UUC UCC GAA CGU GUC ACG UTT-3’. According to the manufacturer's protocol, GC cells were seeded in a 6-well plate, and cultivated for 24h prior to transfection. And transfection was performed when the cells reached ~70% confluence. Subsequent experiments were performed at 48h post-transfection.

### Plasmid construction and transfection

Human ILF3 Gene cDNA Clone (full-length ORF Clone) was cloned into Flag-ILF3 vector. For transfection, the Lipofectamine 3000 and OPTI-MEM (Gibco, Shanghai, China) were mixed with plasmids, transfected into cells and incubate for 24 hours.

### Cell proliferation assay (Edu assay)

GC cells were seeded in 12-well plates to confluence and cultured with 10-μM EdU for an additional 2h. Then 4% formaldehyde (PFA) was used to fix the cells for 30 min at room temperature (RT). After washing, Click-iTR EdU kit was used to detect EdU, with a detection time of ~30 min. After 10 min incubation with DAPI, the cells were observed with a fluorescence microscope (Olympus). The EdU incorporation rate was calculated by Image-Pro Plus 6.0 software (Media Cybernetics). The result was expressed as the ratio of EdU-positive cells to total DAPI-positive cells.

### Flow cytometry for cell cycle analysis

Gastric cancer cells were centrifuged (1000g, 5 min, and 4° C), and rinsed with a volume (1 ml) of precooled PBS. Then a volume (1 ml) of precooled 70% ethanol was added, and the cells were maintained at 4° C for 2 h. The cell suspension was added with a volume (1 ml) of precooled PBS, and the supernatant was discarded after centrifugation (1000 g, 5 min, and 4° C). The cells were resuspended in a volume (500 μl) of binding buffer with 25 μl propidium Iodide (20 x) and 10 μl RNase A (50 x) (RT, darkness, and 30min). Then the flow cytometry was performed.

### Detection of cell migration and invasion

Human gastric cancer cell lines were collected, and suspended with 1640 medium (Gibco), made into 1.5 x 10^5^ cells/ml. The cell suspension (200 μl) was incubated into the upper chamber for migration (or precoated with 100μl Matrigel solution [BD] for invasion). A volume (600 μl) of medium containing 20% FBS and specific treatment was applied to the lower chamber. After 24 h plating, the cells remaining on the upper chamber were removed with a cotton swab. The cells in the lower chamber were fixed and stained. And the number of migration and invasion cells was counted and photographed in three randomly selected view-fields.

### Western blotting

The total proteins were collected from gastric cancer cells grown in a 6-well plate with specific treatment. Briefly, the cells were harvested and lysed with RIPA lysis buffer containing 1× protease inhibitor cocktail. A portion (5 μl) of protein samples was separated by SDS-PAGE, and transferred onto PVDF membrane. PVDF membrane was blocked with 5% non-fat milk (1h, room temperature), and incubated with primary antibody (overnight, 4° C). The proteins on PVDF Membrane were incubated with secondary anti-rabbit antibodies (1h, room temperature). The levels of proteins and phosphoproteins were determined with WesternBright ECL. The primary antibodies were against ILF3 (Abcam, USA), PCNA (Abcam, USA), cyclinD1 (Abcam, USA), MMP9 (Abcam, USA), MMP2 (Abcam, USA), Akt (CST, USA), p-Akt (CST, USA), PI3K (CST, USA), p-PI3K (CST, USA), mTOR (CST, USA), and p- mTOR (CST, USA).

### Immunohistochemistry staining (IHC)

Gastric cancer tissues and adjacent nonmalignant tissues were PFA-fixed, paraffin-embedded, and cut into 5-μm-thick consecutive sections. Each section was then immersed in sodium citrate solution (pH 6.0, 20 min, 98° C) and washed three times with 1x PBS for 3 min/time to achieve deparaffinization and antigen recovery. The sections were permeabilized with PBS containing 0.5% Triton X-100 for 20 min at RT. After initial incubation in 4% normal goat serum (30 min, RT), primary antibody was used and incubated (4° C, overnight). After washing with PBS, each tissue section was incubated with secondary antibody for 1 h at RT, and then stained with diaminobenzidine (DAB). After washing in PBS, nuclei were stained with hematoxylin (Sigma-Aldrich) for 10 min, and washed in tap water. Immunohistochemistry for each sample was repeated thrice.

### Real-time quantitative PCR (RT-qPCR)

RNAfast200 was applied to extract the total RNAs. Reverse transcription was performed in a total volume 20 μl with reverse transcriptase. To remove genomic DNA, samples were mixed with gDNA wipeout buffer and incubated (42° C, 2min), and further incubated at 37° C for 15 min, and 85° C for 5 s to obtain cDNA. The prepared sample was stored at −20° C until use.

The quantification cycle (Cq) was calculated with the amplification curve. The experiments were repeated thrice. The primers for ILF3 were forward 5’-CATTACGCCCATGAAACGCC-3’, and reverse 5’-TAAAGATGGGGGCATGGACG-3’. The primers for GAPDH were forward 5’-GCACCGTCAAGGCTGAGAAC-3’, and reverse 5’-TGGTGAAGACGCCAGTGGA-3’.

### Xenograft tumor

Four-week-old male nude mice were obtained from Sibeifu Company (Beijing, China). A total of 1 x 10^7^ HGC-27 cells were subcutaneously injected into nude mice (n = 5 per group). Nude mice were randomly divided into four groups (n = 5 per group): normal diets, high-fat diets, high-fat diets + statins treatment, and high-fat diets + ILF3 inhibitor-YM155 treatment. The tumor size was measured every 4 days with a vernier caliper starting from the day 7 after subcutaneous tumor. The treatment group were injected with statins (intraperitoneally, 5 mg/kg) or ILF3 inhibitor (intraperitoneally, 5 mg/kg) once every 3 days. The tumor volume was calculated with volume = 0.5 x length x width2. All animal experiments met the ethic regulations.

### Statistical analysis and bioinformatics

SPSS 26.0 and GraphPad Prism 9.1.2 software were used to perform statistical analysis. Data was expressed as mean ± SD of 3 independent biological replicates. Differences between two groups were analyzed by unpaired two-tailed Student's t-test. Multiple comparisons between groups were performed with ANOVA test. A P-value < 0.05 was considered as statistically significance.

### Ethics approval

All investigations conformed to the principles outlined in the Declaration of Helsinki and were performed with permission by the responsible Medical Ethics Committee of Qilu Hospital of Shandong University.

## Supplementary Material

Supplementary Table 1

Supplementary Table 2

Supplementary Table 3
